# Expanded Diversity and Host Range of Bovine Hepacivirus—Genomic and Serological Evidence in Domestic and Wild Ruminant Species

**DOI:** 10.3390/v14071457

**Published:** 2022-06-30

**Authors:** Jana Breitfeld, Nicole Fischer, Ilia Tsachev, Plamen Marutsov, Magdalena Baymakova, Radim Plhal, Oliver Keuling, Paul Becher, Christine Baechlein

**Affiliations:** 1 Institute of Virology, Department of Infectious Diseases, University of Veterinary Medicine Hannover, 30559 Hannover, Germany; jana.breitfeld@tiho-hannover.de; 2 Institute of Medical Microbiology, Virology and Hygiene, University Medical Center Hamburg-Eppendorf, 20251 Hamburg, Germany; nfischer@uke.de; 3 Department of Microbiology, Infectious and Parasitic Diseases, Faculty of Veterinary Medicine, Trakia University, 6000 Stara Zagora, Bulgaria; ilia_tsachev@abv.bg (I.T.); plamen_marutsov@yahoo.com (P.M.); 4 Department of Infectious Diseases, Military Medical Academy, 1606 Sofia, Bulgaria; dr.baymakova@gmail.com; 5 Department of Forest Protection and Wildlife Management, Mendel University, 61300 Brno, Czech Republic; radim.plhal@mendelu.cz; 6 Institute for Terrestrial and Aquatic Wildlife Research, University of Veterinary Medicine Hannover, 30173 Hannover, Germany; oliver.keuling@tiho-hannover.de

**Keywords:** bovine hepacivirus (BovHepV), cattle, wild ruminants, Bulgaria, Germany, Czech Republic, distribution, genetic diversity, host range

## Abstract

The hepatitis C virus (HCV)-related bovine hepacivirus (BovHepV) can cause acute as well as persistent infections in cattle. The true clinical relevance of the virus is not yet known. As reliable antibody detection methods are lacking and prevalence studies have only been conducted in cattle and few countries to date, the true distribution, genetic diversity, and host range is probably greatly underestimated. In this study, we applied several RT-PCR methods and a nano-luciferase-based immunoprecipitation system (LIPS) assay to analyze bovine serum samples from Bulgaria as well as wild ruminant sera from Germany and the Czech Republic. Using these methods, BovHepV infections were confirmed in Bulgarian cattle, with viral genomes detected in 6.9% and serological reactions against the BovHepV NS3 helicase domain in 10% of bovine serum samples. Genetic analysis demonstrated co-circulation of highly diverse BovHepV strains in Bulgarian cattle, and three novel BovHepV subtypes within the genotype 1 could be defined. Furthermore, application of a nested RT-PCR led to the first description of a BovHepV variant (genotype 2) in a wild ruminant species. The results of this study significantly enhance our knowledge of BovHepV distribution, genetic diversity, and host range.

## 1. Introduction

The hepatitis C virus (HCV) is a worldwide cause of acute and chronic human liver disease. Recently, treatment has become possible through the development of direct-acting antivirals (DAAs), but their high costs and limited availability, as well as undiagnosed cases and reinfections, prevent eradication of HCV [1]. For global control, a protective vaccine would be necessary. However, the high genetic diversity of HCV and the lack of an immunocompetent animal model hamper vaccine development [2,3]. Up to 2011, HCV and a distantly related virus of unknown origin termed GB virus B were the only recognized members of the genus *Hepacivirus*, within the family *Flaviviridae*. The positive sense RNA genome of hepaciviruses is characterized by a single open reading frame (ORF), which is flanked by a 5′ and a 3′ untranslated region (UTR). The ORF is translated into a multifunctional polyprotein mediated by an internal ribosomal entry site (IRES) located in the 5′UTR [4]. The polyprotein is cleaved by host and viral proteases into ten distinct proteins, including three structural (Core, E1, E2) and seven non-structural (p7, NS2, NS3, NS4A, NS4B, NS5A, NS5B) proteins [5].

In 2011, a novel hepacivirus was detected in respiratory samples from dogs in the US [6], but later, it turned out that horses are the natural host of this virus [7]. The novel virus was termed non-primate hepacivirus (NPHV) (now referred to as equine hepacivirus, EqHV) and is considered the first indication of expanded diversity of hepaciviruses in non-human hosts. Thus far, the discovery of EqHV has paved the way for the detection of 14 hepaciviral species (*Hepacivirus A-N*) [8] infecting different mammalian hosts including dogs [6], horses [7], non-human primates [9], rodents [10], bats [11], cattle [12,13], squirrels [14], possums [15] sloths [16], shrews [17], and marsupials [18]. In addition, hepaciviruses have been identified in non-mammalian [19,20] and even non-vertebrate [21,22] hosts. Those discoveries of novel HCV-related viruses in animals provided new opportunities for the development of HCV surrogate models [23].

The discovery of diverse hepacivirus sequences in different host species of the orders *Rodentia*, *Chiroptera*, and *Primates* suggests that hepaciviruses do not display a strict host tropism [9,11,24,25]. Furthermore, EqHV has not only been detected in hosts from the family *Equidae*, but also sporadically in dogs [6,7,26]. This broad host range indicates that there are still many unknown animal species harboring these viruses, showing the need to further characterize hepaciviruses in different animal hosts.

The bovine hepacivirus (BovHepV) was first described in 2015 by two separate studies investigating bovine serum samples from Ghana [12] and Germany [13]. Subsequently, it has been detected in cattle from Brazil [27], Italy [28], the US [29], China [30], and Turkey [31], suggesting a worldwide distribution with varying BovHepV RNA detection rates (0.6–14.8%). One study investigated the course of infection in a BovHepV positive dairy herd and demonstrated that BovHepV resembles HCV in its ability to cause acute as well as persistent infections [32]. Acute infection seemed mostly to lead to seroconversion followed by viral clearance. Persistently infected cattle surprisingly showed no marked antibody responses, which stands in contrast to EqHV infections [33]. So far, BovHepV has not been shown to cause liver damage or disease. However, high loads of viral RNA that have been detected in the liver of a BovHepV positive cow and the presence of a micro-RNA (miR)-122 binding site suggest that it also resembles HCV its liver tropism, but the true clinical relevance of the virus remains to be determined [13].

So far, BovHepV is the only member of the species *Hepacivirus N*. Different viral subtypes have been described in several parts of the world over the past years, indicating that the virus is much more widespread and diverse than we know. BovHepV geno- and subtyping is performed according to the HCV classification criteria [8]. A novel genotype is defined if the viral sequence shows <77% identity on the amino acid (aa) level to all other known strains, and a novel subtype must exhibit <85% nucleotide (nt) identity to sequences of the same genotype. So far, two BovHepV genotypes have been described [34,35] and eight subtypes (A-H) have been assigned to the genotype 1 [35,36]. Accordingly, subtypes A and F include two clusters of BovHepV infecting cattle from Germany [37]. Strains from China, Ghana, and Brazil are allocated to subtypes G, E, and H [30,35,36], B and C [12], and D [38], respectively. Genotype 2 includes two recently described sequences from cattle from China [34] and Brazil [39], albeit only a partial coding sequence (CDS) is available for the Brazilian strain.

To date, the diversity and distribution of BovHepV is probably greatly underestimated due to limited sequence information from only a few countries and a lack of serology studies. The present study aimed to further expand our knowledge of the geographical distribution, genetic variability, and host range of BovHepV. To reach those goals, serum samples from Bulgarian cattle were screened for the presence of BovHepV RNA and BovHepV-specific antibodies. Furthermore, specimens from wild ruminants from Germany and the Czech Republic were included. This study demonstrates the presence of highly variable BovHepV strains in Bulgarian cattle, including three hitherto unknown subtypes within genotype 1 as well as the first identification of BovHepV in a wild ruminant species.

## 2. Materials and Methods

### 2.1. Serum Samples

Blood samples from Bulgarian cattle (*n* = 360) were collected in 2018 and were taken from 55 different herds originating from 21 of the total 28 Bulgarian counties. The size of the cattle herds was between 50 and 1800 animals. Two to 69 serum samples were collected per county and one to 23 samples per herd. Animal age ranged between four months and five years. Samples were taken from apparently healthy animals. The study was conducted according to the ethical principles of animal experimentation adopted by the Bulgarian Ministry of Agriculture, Food and Forestry. In addition, blood samples of wild ruminants (*n* = 67) were collected in four different regions of the Czech Republic in 2019. Samples included 14 roe deer (*Capreolus capreolus*), 28 red deer (*Cervus elaphus*), 16 sika deer (*Cervus nippon*), two fallow deer (*Dama dama*), and seven mouflon (*Ovis aries musimon*) samples. Wild ruminant sera from Germany, Lower Saxony, (*n* = 215) were collected during hunts in 2017–2018 and included 160 roe deer, 52 red deer, and three fallow deer samples.

### 2.2. Screening for Viral Genome Fragments in Domestic and Wild Ruminants

For detection of viral RNA, 140 µL of each serum sample was used for RNA isolation with the QIAamp Viral RNA Mini Kit (Qiagen, Hilden, Germany) according to the manufacturer’s protocol. RNA was eluted in 60 µL of Buffer AVE and stored at −80 °C until further use. Bovine serum samples from Bulgaria were tested in pools of five samples each by quantitative reverse transcription (qRT) PCR using the Superscript III One-Step RT PCR System with Platinum Taq Polymerase (Invitrogen, Carlsbad, CA, USA) and primers BovHepV_5NTR_fwd, BovHepV_5NTR_rev, and probe BovHepV_5NTR_probe, as previously described [32]. Individual samples of positive pools were re-tested, and samples with a positive quantification cycle (Cq)-value (<37) were amplified in a pan-hepaci nested RT-PCR, as described previously [32]. The same method was applied to wild ruminant sera. Briefly, cDNA was synthesized from 5 µL of isolated RNA using Superscript II Reverse Transcriptase (RT) (Invitrogen) and 3 µL of reverse transcribed cDNA was amplified in a first PCR with primer pair pan-hepaci_NS3_fwd and pan-hepaci_NS3_rev. Amplification of the PCR product in a second PCR with primer pair pan-hepaci_NS3_nested_fwd and pan-hepaci_NS3_nested_rev gave rise to a 331 nt spanning amplicon. Primers were designed to bind to highly conserved regions of the NS3 helicase domain. PCR was performed using Dream Taq^TM^ Hot Start Green PCR Master Mix (Thermo Fisher Scientific, Waltham, MA, USA) according to the manufacturer’s instructions. PCR products with an expected band size were purified using the GeneJET PCR Purification Kit (Thermo Fisher Scientific) and sent for *Sanger* sequencing (LGC Genomics, Berlin, Germany).

Positive samples from bovines were further amplified with the One Step-RT PCR System with Platinum Taq Polymerase (Invitrogen). Primers BovHepV_3511_fwd and BovHepV_4608_rev have been described previously [32] and gave rise to a 1119 base pairs-spanning amplicon within the NS3 coding region. Products showing the corresponding band size after agarose gel electrophoresis were purified as described and sent for *Sanger* sequencing.

For the pan-hepaci nested RT-PCR positive wild ruminant serum, it was not possible to recover the respective 1119 base pairs spanning amplicon. To solve this problem, the serum sample was subjected to high-throughput sequencing as previously described [12,40], and gaps between sequence information obtained from short read sequencing and *Sanger* sequencing of nested PCR products were filled by semi-nested RT-PCR. This PCR was performed with the Dream Taq^TM^ Hot Start Green PCR Master Mix (Thermo Fisher Scientific) according to the manufacturer’s protocol. Primers used in the semi-nested RT-PCR were designed based on a multiple alignment of BovHepV full-length sequences (GenBank accession numbers KP641123-27, MH027948, MH027953, MN266283-85, MG257793-94, KP265943, KP265946-48, KP265950, MG781018-19, MN691105, MK695669), with special consideration given to genotype 2 sequences (GenBank accession numbers MN691105, MK695669) and on sequence information from *Sanger* sequencing of nested PCR products. They target conserved sequences of the NS3 coding region. Primers used in this study are listed in Table 1.

### 2.3. Next Generation Sequencing and Bioinformatic Analysis

The extracted RNA of three RT-PCR positive bovine serum samples (BovHepV_Bulgaria 9/19/313) and the nested RT-PCR positive red deer serum sample was subjected to next generation sequencing (NGS) analysis. RNA Illumina NGS libraries were prepared from each sample after rRNA removal using the NEBNext rRNA Depletion Kit v2 followed by NEB Ultra II RNA library preparation (New England Biolabs) according to manufacturer’s instructions. Libraries were multiplex-sequenced on an Illumina MiSeq instrument (300 cycles, PE protocol) with approximately 4,000,000 reads per sample. Bioinformatic analysis of the obtained short read files was performed as previously published using our inhouse Pathogen Detection Tool DAMIAN [40].

### 2.4. Phylogenetic Analysis

Sequences obtained after *Sanger* sequencing of RT-PCR (bovine sera, *n* = 17) and semi-nested RT-PCR (wild ruminant serum, *n* = 1) products were trimmed to 835 nt using BioEdit 7.2.5. [41]. The two full-length sequences obtained through NGS analysis were truncated to the ORF coding region. Newly described sequences were aligned with BovHepV full-length and partial NS3 coding sequences downloaded from GenBank using the ClustalW multiple alignment tool implemented in BioEdit. Maximum likelihood trees were constructed in MEGAX version 10.2.0 based on the general time reversible model [42]. Bootstrap analysis was performed with 1000 replicates.

### 2.5. Luciferase Immunoprecipitation System (LIPS) Assay

Serologically reactive serum samples were detected by LIPS. To improve the previously described assay [7,32], the so far used renilla luciferase was replaced by a nano luciferase. For this, the NS3 helicase domain of BovHepV was amplified with primers—pREN-fwd (5′ GAACAAGGATCCGTTTGTACC 3′), including a BamHI-restriction site (underlined) and—pREN-rev (5′AAACTCGAGTCA**AGATCCCTTGTCATCGTCGTCCTTGTAGTCCAT**ATTACAGTCAGTCACACTGTC 3′), adding an N-terminally linked FLAG Tag (bold face) and an XhoI-restriction site (underlined). PCR was performed using Phusion^TM^ High-Fidelity DNA Polymerase (Thermo Fisher Scientific). After digestion of the PCR product and the vector pcDNA 3.1-IL6-secNLuc (kindly provided by Imke Steffen, University of Veterinary Medicine Hannover) with respective restriction enzymes, the linearized vector and insert BovHepV-NS3frag-FLAG were ligated with T4 DNA ligase (Thermo Fisher Scientific). Selection and growth of ampicillin resistant *E. coli* Top 10 clones (Thermo Fisher Scientific) was performed in LB medium, and successful cloning was confirmed through subsequent *Sanger* sequencing.

For transfection, 1.5 × 10^6^ Cos-1 cells were plated in a 10 cm dish. The next day, cells were transfected with 24 µg of plasmid using Lipofectamine^TM^ 2000 Transfection Reagent (Invitrogen). Empty vector pcDNA 3.1-IL6-secNLuc was transfected simultaneously as control. After 48 h of incubation, the supernatant was harvested, centrifuged twice for 4 min at 14.000× *g* and 4 °C, and stored in aliquots at −80 °C. Concentrations of Light Units (LU) per µL of supernatant were measured using NanoGlo Luciferase Assay System (Promega, Madison, WI, USA) on a plate reader (TriStar Berthold, Bad Wildbad, Germany). Successful expression of the recombinant BovHepV NS3 helicase domain was confirmed by Western Blot analysis based on incubation with monoclonal anti-flag M2 antibody (Sigma Aldrich, St. Louis, MO, USA).

LIPS assay was performed according to previously described protocols [7,32] with some modifications. Briefly, 10 µL of a 1:100 serum dilution were added to 40 µL of buffer A (50 mM Tris, 100 mM NaCl, 5 mM MgCl2, 1% Triton, pH: 7.5) on a transparent 96-well plate (Thermo Fisher Scientific), and an equivalent of 1 × 10^6^ nano luciferase LU was added to each well with a final volume of 50 µL. After 1 h of incubation, the antibody–antigen mixture was incubated with and captured by protein A/G beads (Thermo Fisher Scientific) on a filter plate (Merck Milipore, Molsheim, France). Free complexes were removed by washing the plate (4× with buffer A; 1× with PBS). Relative Light Units (RLU) were measured in a 5 s read after addition of the NanoGlo substrate furimazine (diluted 1:50 in NanoGlo buffer). Serum samples were tested in duplicates, and the mean was used for calculation of a sample to positive (S/P) ratio. Monoclonal anti-flag M2 antibody (Sigma Aldrich) and a bovine serum previously determined to be positive for anti-NS3 antibodies (serum 448) were included as positive controls in each run. In addition, cells containing no or previously determined negative serum or supernatant of cells transfected with the control plasmid were used as negative controls. To determine a cut-off value, the S/P ratio (value of each sample divided by the value of positive control serum 448) was calculated for 215 known negative sera. A fivefold standard deviation was added to the mean to define a positive sample. Inter- and intra-assay coefficients of variability (CV) were calculated to describe plate-to-plate consistency and variations of results within one experiment.

## 3. Results

### 3.1. Identification and Genetic Characterization of BovHepV in Bulgarian Cattle

**Figure 1:**
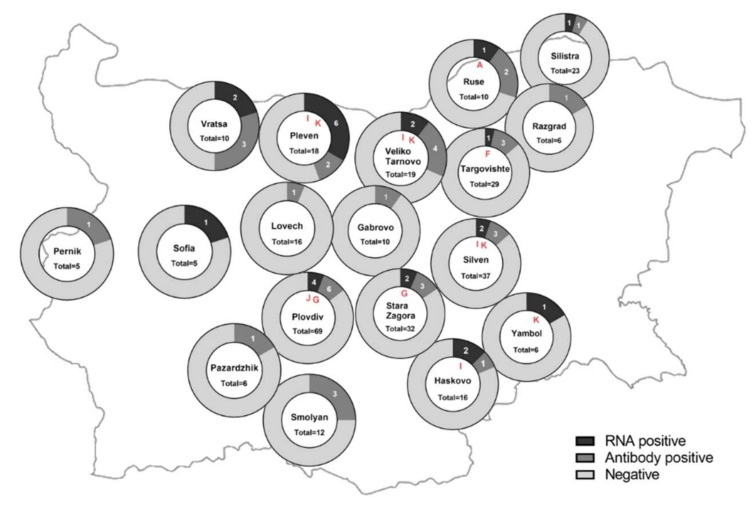
Detection rates of BovHepV genomes and BovHepV anti-NS3 antibodies in different regions of Bulgaria. The total number of investigated samples, the number of RNA positive and serologically reactive samples and the respective geographical origin are shown. The circulating BovHepV subtypes are depicted in the central circle and marked in red. The map was downloaded from GinkgoMaps [http://ginkgomaps.com accessed on 1 January 2022] and the illustrations were created with GraphPad Prism.

Of 360 bovine serum samples tested, 36 (10%) had a positive Cq value of <37 in the RT-qPCR assay and were tested by further PCR methods. In total, 25/360 (6.9%) samples were positive for viral RNA in the pan-hepaci nested PCR and/or the RT-PCR and were considered as positive for BovHepV RNA. Positive samples originated from 15 different herds (27%) that were located in twelve different counties (43%) distributed over the country (Figure 1, see also Table S1). In the vast majority of cases, one or two animals per herd were RNA positive. However, in one farm, viral genomes were found in six out of a total of twelve tested animals (50%).

**Figure 2:**
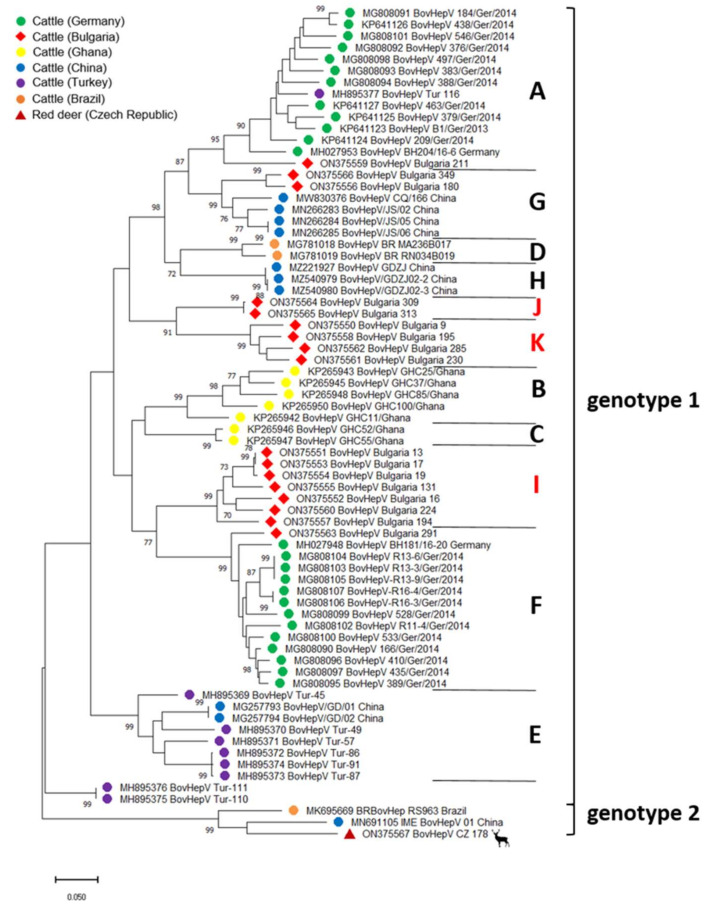
Maximum Likelihood phylogenetic tree based on partial NS3 coding sequences (835 nt) of hepaciviruses in ruminant species. Bootstrap values <70% are not shown. BovHepV subtypes (in capitals) and genotypes are indicated on the right. Color codes of BovHepV sequences specify the country of origin. The newly discovered Bulgarian sequences are marked with a red rhombus and the newly described subtypes I–K are depicted in red letters on the right side. The newly discovered red deer sequence is marked with a red triangle. Scale bar indicates nucleotide substitutions per site.

The generation of a 1119 base pairs-spanning amplicon through RT-PCR and subsequent *Sanger* sequencing was possible in 17/25 RNA positive samples. Samples were trimmed to 835 nt within the highly conserved NS3 coding region for phylogenetic analysis. All sequences were submitted to GenBank under accession numbers ON375550-ON375566. Comparative analysis with available BovHepV sequences demonstrated high genetic diversity among different BovHepV strains detected in Bulgarian cattle. Sequence identities among newly discovered strains ranged from 79.0 to 99.9% on the nucleic acid level and from 92.5 to 100.0% for the deduced amino acid sequences. Amino acid identities with sequences from genotype 1 were high (82.9–98.6%), while identities with genotype 2 sequences ranged between 78.2 and 82.9%. A phylogenetic tree based on the partial NS3 coding sequences determined in this study and all respective sequences available at GenBank revealed that the sequences of only four BovHepV strains from Bulgaria were assigned to previously described subtypes of BovHepV genotype 1 (Figure 2). One of those partial NS3 sequences (BovHepV_Bulgaria_291) was most closely related to BovHepV strains from Germany belonging to subtype F (>91.7% nt identity). Sequences BovHepV-Bulgaria_180 and 349 were obtained from cattle from two different herds and shared 96.0% nt identity among each other but <84.2% to all other sequences from Bulgaria. They were most closely related to strains from China representing BovHepV subtype G with >90.2% nt identity. Another sequence (BovHepV_Bulgaria_211) exhibited high similarities with subtype A sequences (>86.8% nt identity), including strains from Germany. Assignment to either subtype F, G, or A was supported by high bootstrap values (Figure 2).

In contrast, the other 13 newly described sequences formed three additional, hitherto unknown subtypes within BovHepV genotype 1 (Figure 2). The presence of those three novel distinct subtypes based on analysis of partial NS3 coding sequences prompted us to determine complete polyprotein coding sequences (CDS) from one representative of each cluster through NGS analysis. Full-length sequences could be obtained for strains BovHepV_Bulgaria_19 (novel subtype I, see below) and 9 (novel subtype K, see below). Sequences were submitted to GenBank under the following accession numbers: ON402464 and ON402465. We failed to recover a full-length sequence for the strain BovHepV_Bulgaria_313 (proposed subtype J), probably due to the bovine sample’s low amounts of viral RNA (Cq: 31.1).

**Figure 3:**
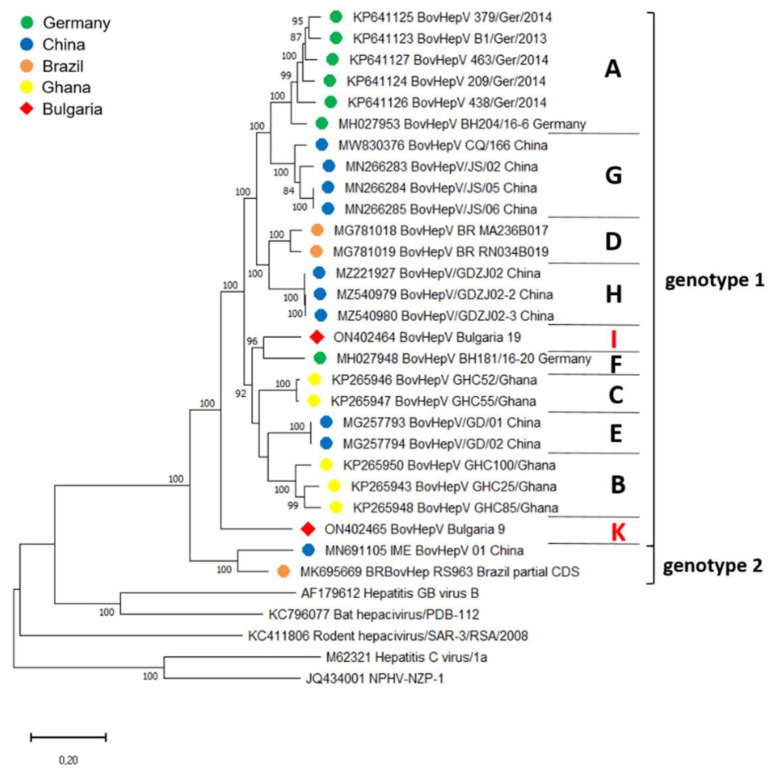
Maximum Likelihood phylogenetic tree based on complete BovHepV polyprotein coding sequences deposited in GenBank. Only a partial sequence of 6102 nucleotides has been reported for BovHepV sequence BRBovHep RS963 Brazil. Bootstrap values <70% are not shown. BovHepV subtypes (in capitals) and genotypes are indicated on the right. Color codes of BovHepV sequences specify the country of origin. The newly discovered Bulgarian sequences are marked with a red rhombus and the newly described subtypes I and K are depicted in red letters on the right side. Scale bar indicates nucleotide substitutions per site.

Analysis of the complete CDS of BovHepV_Bulgaria_19 revealed an aa identity of 83.9–95.6% with BovHepV full-length sequences from genotype 1, including the sequence of BovHepV_Bulgaria_9. Aa identity with IME_BovHepV 01 China, representing genotype 2, was 76.9%. Since assignment to a BovHepV genotype requires an aa identity of >77% with other sequences of the same genotype, strain BovHepV_Bulgaria_19 was classified as a BovHepV genotype 1 virus. However, nucleotide identities with other full-length sequences from genotype 1 were rather low (74.8–81.6%). As a distinct BovHepV subtype is defined by displaying <85% nt identity with other sequences, the newly described Bulgarian strain represents a novel BovHepV subtype within the genotype 1. Furthermore, BovHepV_Bulgaria_9 represents a novel BovHepV subtype within the genotype 1 as well, exhibiting aa identities of 83.1–84.5% and 73.3% with sequences from genotype 1 and 2, respectively, and nt identities of 73.8–75.6% with remaining genotype 1 strains. Those hitherto unknown BovHepV subtypes were designated subtype I and K. Phylogenetic analysis confirmed the presence of two novel subtypes: the complete coding sequences of the two Bulgarian BovHepV strains were grouped in the common branch of bovine hepacivirus genotype 1, but were clearly distinct from the previously established BovHepV subtypes A–H (Figure 3).

According to the phylogenetic tree based on partial NS3 coding sequences (Figure 2), subtype I included seven sequences sharing 97.9–100.0% aa and 91.5–99.9% nt sequence identity among each other. Those BovHepV strains originated from four different cattle herds located in different regions of Bulgaria (Figure 1). Four of the sequences were obtained from the same herd and shared 100.0% aa identity. Moreover, three of them exhibited nearly complete identity on the nt level as well (99.3–99.9%). Subtype K was represented by four sequences that showed identities of 98.2–99.6% on the aa level and 92.5–94.6% on the nt level. These sequences were obtained from four herds located in four different counties (Figure 1). Surprisingly, in three herds, animals infected with both subtype I and subtype K variants were found (see also Table S1), suggesting co-circulation of different BovHepV subtypes in one herd. The genetic cluster including strain BovHepV_Bulgaria_313, for which no full-length sequence could be generated, included two variants from two animals from the same herd sharing 100.0% aa and nearly complete nucleotide sequence identity (99.8%). Nucleotide identities were 79.4–84.4% and 74.6–75.7% with other sequences of BovHepV genotype 1 and 2, respectively, strongly suggesting that the two variants represent yet another novel BovHepV subtype within the genotype 1, proposed as subtype J.

In the course of genetic typing of the newly described Bulgarian variants, genotyping of BovHepV sequences from Turkey previously described by Yeşilbağ et al. was reconsidered [31]. Consistent with the previous study, strain BovHepV_Turkey_116 was most closely related to subtype A sequences from Germany (Figure 2). Six other sequences from Turkey were grouped with BovHepV subtype E variants from China that have not been included in the previous analysis by Yeşilbağ et al. [31]. Sequences of BovHepV_Turkey_110 and BovHepV_Turkey_111 were only distantly related to other sequences (Figure 2). Comparison of nt and aa identities in the partial NS3 coding region implies that they represent yet another novel BovHepV subtype within the genotype 1 or even another distinct genotype.

### 3.2. Identification and Genetic Characterization of BovHepV in Wild Ruminants

Analysis of a total of 282 serum samples from wild ruminants (14 roe deer, 28 red deer, 16 sika deer, two fallow deer, and seven mouflons from the Czech Republic, as well as 160 roe deer, 52 red deer, and three fallow deer from Germany) by the pan-hepaci nested PCR [32] identified one BovHepV RNA positive sample obtained from a red deer in the Czech Republic (see also Figure S1, Table S2). According to our knowledge, this is the first identification of a hepacivirus in a wild ruminant species. A partial NS3 coding sequence (835 nt) was generated by semi-nested PCR (GenBank accession number ON375567). Comparative sequence analysis revealed only moderate aa homologies to BovHepV genotype 1 sequences (87.8–91.8%), but significantly higher aa identities with BovHepV strains from genotype 2 (97.8–98.2%). Nt identities with genotype 1 and genotype 2 strains were 72.8–76.4% and 83.1–83.2%, respectively. Accordingly, the hepacivirus sequence from red deer is most closely related to BovHepV genotype 2 sequences. The allocation of the red deer hepacivirus sequence to BovHepV genotype 2 is confirmed by the phylogenetic tree based on the partial (835 nt) NS3 coding region (Figure 2). An additional 693 nt comprising fragment encoding a C-terminal part of E1 and an N-terminal part of E2 was obtained through NGS. These data were deposited under GenBank accession number ON871823. Here, nt identities with genotype 1 sequences were even lower (61.3–63.6%), while analysis of this genomic region showed an identity with the sequence of IME _BovHepV_01 China of 80.6%, suggesting that the hepacivirus from red deer represents a novel BovHepV subtype within genotype 2.

### 3.3. Luciferase Immunoprecipitation System (LIPS) Assay

**Figure 4:**
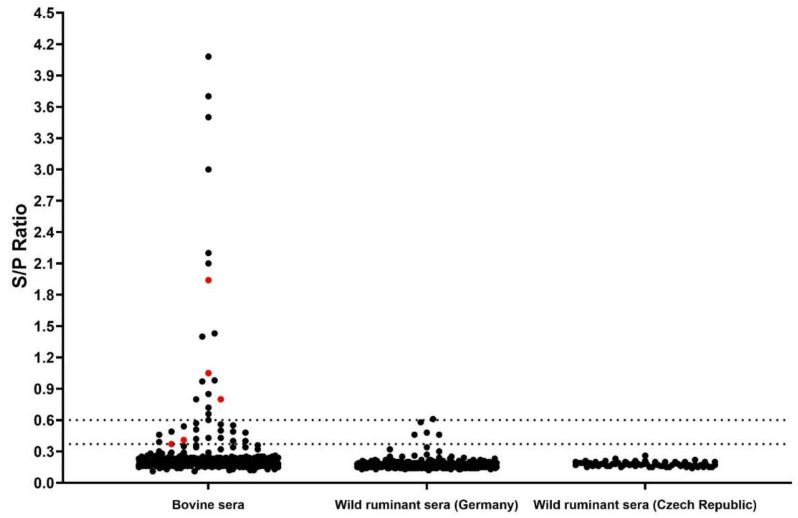
Serological screening of ruminant serum samples through LIPS. Samples are represented by black dots and included bovine sera from Bulgaria (*n* = 360) and wild ruminant sera from Germany (*n* = 215) and the Czech Republic (*n* = 67). The dashed lines represent the cut off values to define negative, moderately and highly reactive samples. Serologically reactive samples positive for BovHepV RNA are marked in red.

All samples from cattle and wild ruminants were screened for anti-BovHepV-NS3 antibodies through a nano-luciferase based LIPS assay. Analysis of cell culture supernatant by Western Blot confirmed successful expression of the recombinant nano luciferase -BovHepV NS3 helicase domain protein (results not shown). Calculation of inter- and intra-assay consistency resulted in a coefficient of variability of 9% in both cases. A cut off value to define a positive sample was set at an S/P ratio of 0.37 as described above. Serological reactions were classified as negative (S/P < 0.37), moderate (S/P 0.37–0.6), and high (S/P > 0.6). Moderately and highly reactive samples were considered seropositive. Altogether, 36 individual bovine samples (10%), corresponding to 24 different herds (43%), displayed a serological reaction. Of those, 18 samples showed a moderate and another 18 samples a high serological reaction against the recombinantly expressed NS3 helicase domain (Figure 4). Respective cattle herds were located in 16 different Bulgarian counties (Figure 1). Five samples were positive for both viral RNA and antibodies. Of those, three displayed a high serological reaction measured by LIPS (Figure 4). Concerning the wild ruminant sera, four moderate and one high serological reaction were observed in four roe and one red deer from the state of Lower Saxony in Germany (Figure 4, see also Figure S1, Table S2); the strongly reactive sample showed an S/P ratio just slightly above the cut-off value to define a highly reactive sample (S/P: 0.61). All serum samples from wild ruminants of the Czech Republic tested negative.

## 4. Discussion

In recent years, knowledge on hepaciviral diversity and distribution has expanded greatly due to novel discoveries in several animal species and geographical areas. A high diversity has been described for BovHepV, with discovery of two genotypes and eight subtypes (A-H) within genotype 1 to date. The various BovHepV strains have been detected in seven countries located on five continents, indicating a worldwide distribution in cattle [12,13,27,29,35]. In the relatively short time span since the first description in 2015, new discoveries of novel BovHepV strains have continued to be described, demonstrating that the true distribution and diversity is far from known. Here, we show the presence of three novel BovHepV subtypes (subtype I, J, and K) in addition to the previously established subtypes A, F, and G in cattle from Bulgaria, a country not previously studied. Initial discovery of BovHepV in cattle indicated only low-to-moderate genome sequence diversity of BovHepV strains in Germany [12]. Shortly thereafter, however, an expanded genetic diversity of BovHepV was reported by Schlottau and colleagues, who showed that two BovHepV subtypes (A and F) circulate in Germany, which differ up to 20% on the nt level [37]. Different clusters of BovHepV among cattle from the same country have also been detected in Ghana [13], China [30,34,35,36], and Brazil [38,39]. In both latter countries, the presence of two highly distinct BovHepV genotypes has been described. Thus, the finding of highly diverse BovHepV strains in cattle from Bulgaria fits into this picture. In this study, two novel subtypes (subtype I and K) were identified based on the analysis of complete polyprotein coding sequences, while one subtype (subtype J) was proposed based on the analysis of a partial NS3 coding region. Among different BovHepV proteins, NS5B and NS3 are highly conserved, and phylogenetic analysis of those regions mostly reflects whole genome analysis [43]. Therefore, the classification described here probably mirrors the true genetic relationship of the different BovHepV variants.

Four of the partial NS3 coding sequences described in this study showed closest relationships to formerly described BovHepV strains from genotype 1 subtypes A, F, and G, including German and Chinese variants. It is not known whether the animals were born and raised in Bulgaria. Thus, it is also possible that the animals were imported from other countries. Similar results have been observed for bovine samples from Turkey [31]. In the mentioned study, one sequence found in an animal that was imported from Italy did not cluster with other Turkish variants, but was closely related to BovHepV subtype A sequences from Germany. Another explanation would include the possibility that BovHepV subtypes A, F, G, and also E (with BovHepV representatives from Turkey and China) do not only circulate in the country where they were first discovered, but are present worldwide. Continuous analysis of circulating BovHepV strains needs to be conducted to prove this hypothesis.

So far, a BovHepV prevalence ranging from 0.6 to 14.8% (mean ~ 7%) has been described in different countries. In the present study, viral genomes were detected in 6.9% of animals. The 25 viral genome-positive cattle originated from 15 different herds (27%) distributed all over Bulgaria. However, it appears that the BovHepV RNA positive cattle farms are located mainly in the north (border with Romania) and southwest (borders with Greece and Turkey) of Bulgaria. Similar husbandry and handling of animals or frequent importation of cattle could be reasons for the spread of highly diverse BovHepV variants in Bulgaria and Turkey. For future studies, it would be interesting to also investigate the infection status of Romanian and Greek cattle to determine if BovHepV is widespread throughout the Mediterranean area and if cross-country transmission might play a role.

In one Bulgarian cattle herd, six out of twelve tested animals were positive for BovHepV RNA. Four of the characterized sequences belong to the newly described BovHepV subtype I and showed high genetic identities in the partial NS3 coding sequence (91.9–99.9% identity), which is indicative of intra-herd transmission of the virus. For the two remaining variants, no partial NS3 sequence could be generated through RT-PCR in one case, while the other sequence belonged to the novel BovHepV subtype K and showed only moderate nt identities to the above-mentioned sequences (79.4–80.6%). Transmission routes of BovHepV are not yet fully understood. So far, there was no evidence for excretion via feces, milk, urine, or nasal discharge, but due to a limited number of tested animals, those routes cannot be excluded [12,30]. HCV is transmitted primarily through contact with blood from infected individuals, with intravenous drug use being the most common cause of infection [44]. In addition, parenteral transmission also appears to be the main route of transmission for EqHV in horses [33,45]. It is likely that similar routes also play a role in BovHepV transmission, e.g., through use of contaminated vaccine needles, providing a possible explanation for the high prevalence observed in the mentioned herd. Vertical transmission of HCV occurs in approximately 5% of infected mothers [46]. This route of virus transfer has also been described for EqHV [47,48] and is well known for other members of the *Flaviviridae*, such as bovine viral diarrhea virus (BVDV) [49]. Accordingly, it is reasonable to speculate that mother-to-fetus infection could play a role in BovHepV epidemiology. So far, only a small number of calves from a BovHepV positive herd were tested with negative results, but evidence of BovHepV RNA in commercial fetal bovine serum supports this assumption [12,29].

In addition to screening for viral genomes, a nano-luciferase based LIPS assay was applied to detect BovHepV specific anti-NS3-antibodies. Serology has the advantage of detecting not only active but also resolved infections. To detect antibodies directed against genetically diverse BovHepV variants, an antigen encoded by the highly conserved NS3 helicase domain region was applied. Humoral immune response against this antigen has been previously demonstrated for infections with HCV [50] and EqHV [7]. To date, only one serological study has been conducted on BovHepV, using a renilla-luciferase based LIPS assay [32]. We decided to use the nano-luciferase due to its brighter, more stable, and more sensitive signal. With this assay, we found a seroprevalence of 10% on the individual and 43% on the herd level (Figure 4). In previous studies, detection rates of EqHV and BovHepV anti-NS3 antibodies were higher in horses (>30%) [7,33,51,52,53,54,55,56] and in cattle from Germany (19.9%) [32], respectively, compared to our findings. Application of highly conserved NS3 helicase domain as antigen ensures binding of serum antibodies induced even through highly diverse BovHepV variants. Thus, the seroreactivity determined in the present study most likely reflects the infection status of Bulgarian cattle. RT-PCR and serology both confirmed that BovHepV infections are widespread throughout the country.

In our study, the presence of viral RNA was confirmed in only three highly serological reactive samples. This might indicate that the humoral immune response is involved in viral clearance. This would be consistent with a previous study, which showed that acutely infected cattle mostly displayed a clear antibody response with subsequent viral clearance [32]. Acute infections with seroconversion and viral clearance also seem to be the predominant course of infection for EqHV in horses [33,57]. Thus, both viruses appear to differ from HCV in the ability of their hosts to efficiently clear the virus, which might also be the reason that no or only minor liver damage is seen in infections with animal hepaciviruses.

So far, BovHepV has only been detected in cattle, although recently, in China, BovHepV RNA has been identified in ticks sucking blood on cattle [36]. Nevertheless, the authors of the mentioned study suspected the sequences to be derived from the ticks’ vertebrate host. A broader host range has been described for rodent, bat, and primate hepaciviruses [9,11,24,25] and is even exceeded by EqHV infections, which have not only been detected in horses, donkeys, and mules, but also sporadically in dogs [6,7,26,52,58,59]. The origin of EqHV in dogs is not fully understood. Both direct cross-species transmission events as well as transmission via feeding of animals with horse meat or application of horse-serum derived veterinary products were suggested [60]. A cross-species transmission from horses to donkeys or mules, likely facilitated by the close genetic relatedness of the host species, is very likely [26]. One limiting factor for viruses to switch hosts is the host’s immune response. Hepaciviruses are able to evade the innate immune response by cleavage of the mitochondrial antiviral signaling protein (MAVS) via their NS3/4A protease. This strategy of immune evasion is conserved among hepaciviruses [61], allowing it in principle to cross species barriers. Serological reactivities of porcine, equine, and human serum samples against the BovHepV NS3 helicase domain were analyzed previously and showed weak reactions in horses and pigs, whereas one pig showed high serological reactivity [32]. However, it appears likely that such reactions have resulted from infection with related hepaciviruses.

If BovHepV can infect hosts other than cattle, it is likely that other ruminant species would be affected. This consideration led us to look at the wild ruminant population and to examine serum samples taken on hunts in Germany and the Czech Republic. In fact, one red deer from the Czech Republic tested positive for BovHepV RNA and genetic analysis confirmed the presence of the recently described BovHepV genotype 2 in this blood sample [34]. To our knowledge, this is the first identification of a BovHepV infection in a wild ruminant species. At this time, it can only be speculated if this resulted from cross-species transmission events between domestic and wild ruminants or whether our finding is indicative of an independent wildlife infection cycle. To pursue this further, it would also be necessary to analyze bovine serum samples from the Czech Republic and wild ruminants from other origins for the presence of BovHepV.

Surprisingly, no clear serological reactivity of wild ruminant sera was observed in the LIPS assay. Three roe deer and one red deer sample from Germany displayed moderate serological reactions and one roe deer sample from Germany exhibited luciferase units just above the cut-off value for defining a highly reactive sample (S/P 0.61). It can only be speculated because there are only a few and rather weak serological reactions in wild ruminants. As it is generally not known how the immune system of those animals reacts to BovHepV infections, one possible explanation might be that only antibodies with low affinity are produced. Since little evidence of BovHepV infections in wild ruminants has been detected in the present study, it can be assumed that cattle represent the natural host of the virus. Sporadic spillover from cattle into the wildlife population and subsequent adaptation to the new host could be another possible explanation for lower viral replication in wild ruminants. Nevertheless, transmission from wild ruminants into the cattle population cannot be ruled out and is supported by the basal clustering of the red deer hepacivirus in the phylogenetic tree. Further identification and characterization of BovHepV RNA in wild ruminants would be needed to clarify the virus transmission pathways between domestic and wild ruminant species. In both cases, additional indirect transmission routes would be required for the virus to cross species, e.g., through contaminated feed or water or through the fecal-oral route.

To conclude, we identified highly diverse BovHepV strains in cattle from Bulgaria and defined three novel BovHepV subtypes (I, J, and K) within genotype 1. Furthermore, we found a diverse BovHepV strain in a red deer from the Czech Republic and demonstrated for the first time that BovHepV is also able to infect ruminant species other than domestic cattle.

## Figures and Tables

**Table 1:** Overview of primers used in this study to detect and characterize BovHepV RNA.

Primer/Probe	PCR Assay	Animal Species	Sequence (5′-3′)	Source
BovHepV_5NTR_fwd	RT-qPCR	Bovine	AACAGGCCCCTAGTAG	Baechlein et al. [32]
BovHepV_5NTR_rev	RT-qPCR	Bovine	GTACTCGGTCCTTCCCA	Baechlein et al. [32]
BovHepV_5NTR_probe	RT-qPCR	Bovine	CATGAGCCCTTTCCCCACAGATTGAGTGGA	Baechlein et al. [32]
Pan-hepaci-NS3_fwd	Nested RT-PCR	Bovine	GCMCCTACKGGSTCYGGGAA	Baechlein et al. [32]
Pan-hepaci-NS3_rev	Nested RT-PCR	Bovine	TCRAAGTTCCCRGTGTAMCCMGTCAT	Baechlein et al. [32]
Pan-hepaci-NS3_nested_fwd	Nested RT-PCR	Bovine	GAYGTGRTCATYTGTGATGARTGCCA	Baechlein et al. [32]
Pan-hepaci-NS3_nested_rev	Nested RT-PCR	Bovine	CCSCGATAGTARGCSACWGC	Baechlein et al. [32]
BovHepV_3511_fwd	RT-PCR	Bovine	TGGGARGTCCARACTGTCTATG	Baechlein et al. [32]
BovHepV_4608_rev	RT-PCR	Bovine	CTCATAACARATCTGCCTCTGC	Baechlein et al. [32]
CZ178_semi-nested_fwd_1	Semi-nested RT-PCR	Wild ruminants	ATGTCGGCGATCTCAACTTCC	This study
CZ178_semi-nested_fwd_2	Semi-nested RT-PCR	Wild ruminants	ACACTGTGAGAGTCTCGCAGC	This study
CZ178_semi-nested_rev_1	Semi-nested RT-PCR	Wild ruminants	TGAGCACGAACGACGCTGTTG	This study
CZ178_semi-nested_fwd_3	Semi-nested RT-PCR	Wild ruminants	CATGGTGCARCGGTGCAG	This study
CZ178_semi-nested_fwd_4	Semi-nested RT-PCR	Wild ruminants	ATGCAYTATGTCCGAAAGGGC	This study
CZ178_semi-nested_rev_2	Semi-nested RT-PCR	Wild ruminants	AGTAGCTGTGGCAAGCAGAAC	This study

## Data Availability

The sequencing data presented in this study are available in GenBank under accession numbers ON375550-ON375567, ON402464, ON402465, and ON871823.

## Supplementary Materials

The following supporting information can be downloaded at: https://www.mdpi.com/article/10.3390/v14071457/s1, Figure S1: Detection rates of BovHepV genomes and BovHepV anti-NS3 antibodies in wild ruminants from Germany and the Czech Republic, Table S1: Overview of investigated Bulgarian cattle farms, Table S2: Overview of sampling sites and detection of BovHepV RNA and anti-NS3 antibody positive samples from wild ruminants in Germany and the Czech Republic, Table S3: Estimates of Evolutionary Divergence between partial NS3 Sequences, Table S4: Estimates of Evolutionary Divergence between Complete Polyprotein Coding Sequences.
