# Supplementary material for: Expanded Diversity and Host Range of Bovine Hepacivirus—Genomic and Serological Evidence in Domestic and Wild Ruminant Species
Source: Viruses. 2022 Jun 30;14(7):1457. doi: 10.3390/v14071457 (PMC9319978; doi:10.3390/v14071457)
Supplement: Supplementary file 1 [file viruses-14-01457-s001.zip › Figure S1.pdf]

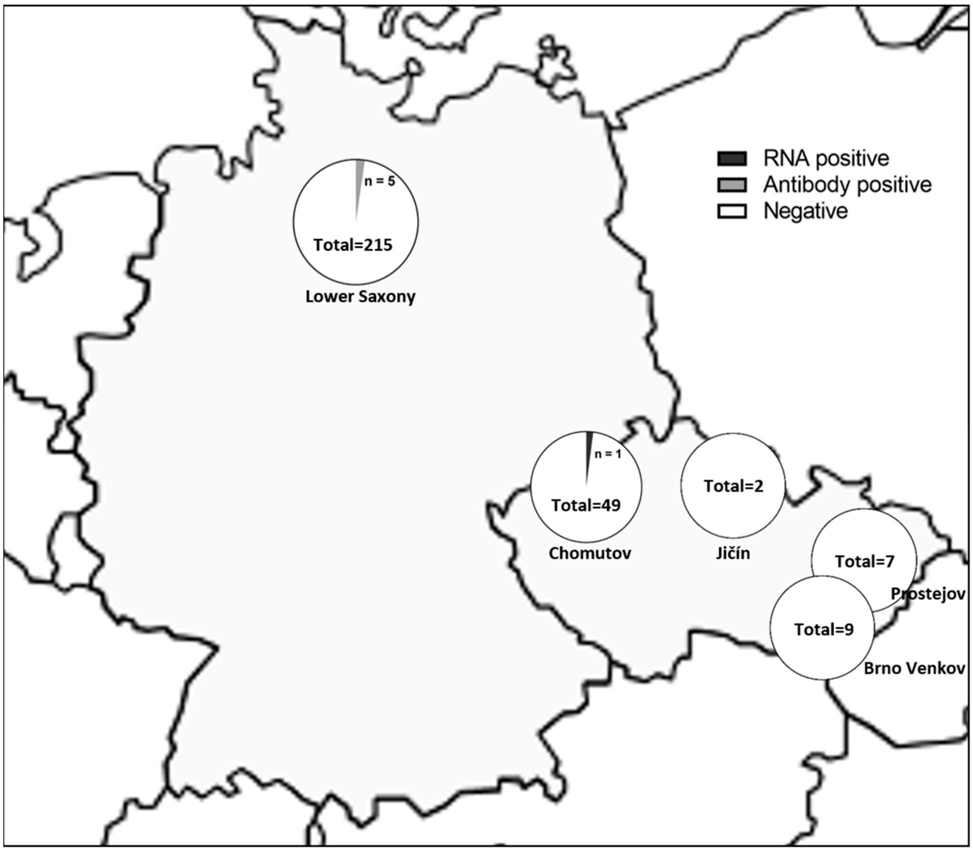

Figure S1: Detection rates of BovHepV genomes and BovHepV anti-NS3 antibodies in wild ruminants from Germany and the Czech Republic. The total number of investigated samples, the number of RNA positive (black) and serologically reactive samples (grey) and the respective geographical origin are shown. Illustrations were created with Graph PadPrism.
