# Supplementary material for: Expanded Diversity and Host Range of Bovine Hepacivirus—Genomic and Serological Evidence in Domestic and Wild Ruminant Species
Source: Viruses. 2022 Jun 30;14(7):1457. doi: 10.3390/v14071457 (PMC9319978; doi:10.3390/v14071457)
Supplement: Supplementary file 1 [file viruses-14-01457-s001.zip › Table S 1.pdf]

Table S 1. Overview of investigated Bulgarian cattle farms. The approximate herd size, numbers of analyzed serum samples, as well as RNA and antibody positive animals are given. For BovHepV positive herds, circulating viral subtypes are specified.

Bulgarian County	Cattle Farms (id Number)	Herd Size	Total Number of Sampled Animals per Herd	Number of RNA Positive Animals	BovHepV Subtype	Number of Antibody Positive Animals
Blagoevgrad	1	54	2	-		-
Dobrich	2	1753	5	-		-
	3	143	4	-		-
	4	161	5	-		-
	5	241	5	-		-
	6	230	1	-		-
Gabrovo	7	377	5	-		-
	8	73	5	-		1
Haskovo	9	883	8	-		-
	10	422	6	2	I	1
	11	762	2	-		-
Lovech	12	218	4	-		-
	13	397	8	-		1
	14	228	4	-		-
Pazardzhik	15	89	6	-		1
Pernik	16	371	5	-		1
Pleven	17	284	12	6	I, K	2
	18	63	4	-		-
	19	164	2	-		-
Plovdiv	20	467	19	1	J	-
	21	817	18	2		3
	22	118	1	-		-
	23	146	5	-		1
	24	132	3	-		-
	25	257	9	-		1
	26	129	4	-		-
	27	409	7	-		1
	28	83	3	1	G	-
Razgrad	29	323	3	-		-
	30	114	3	-		1
Ruse	31	211	10	1	A	2
Shoumen	32	394	3	-		-
Silistra	33	363	23	1		1
Silven	34	677	20	2	I, K	2
	35	578	12	-		1
	36	341	5	-		-
Smolyan	37	1825	5	-		-
	38	208	7	-		3
Sofia	39	173	4	1		-
Stara Zagora	40	64	2	-		-
	41	179	18	-		-
	42	82	7	1	G	2
	43	62	5	1		1
Targovishte	44	56	2	-		1
	45	481	21	1	F	1
	46	184	6	-		1
Varna	47	296	7	-		-
Veliko Tarnovo	48	unknown	1	-		-

	49	unknown	2	-		-
	50	unknown	2	-		-
	51	94	3	-		-
	52	337	7	2	I, K	3
	53	408	4	-		1
Vratsa	54	219	10	2		3
Yambol	55	72	6	1	K	-
