# Supplementary material for: Expanded Diversity and Host Range of Bovine Hepacivirus—Genomic and Serological Evidence in Domestic and Wild Ruminant Species
Source: Viruses. 2022 Jun 30;14(7):1457. doi: 10.3390/v14071457 (PMC9319978; doi:10.3390/v14071457)
Supplement: Supplementary file 1 [file viruses-14-01457-s001.zip › Table S 2.pdf]

Table S 2: Overview of sampling sites and detection of BovHepV RNA and anti-NS3 antibody positive samples from wild ruminants in Germany and the Czech Republic. The total number of sampled animals, the host species (*Capreolus capreolus*, CC, *Cervus elaphus*, CE, *Cervus nippon*, CN, *Dama dama*, DD, *Ovis aries musimon*, OM) and numbers and hosts of RNA and antibody positive wild ruminants are given. For RNA or antibody positive wild ruminants, the sex of the animals is specified

Sampling Location	Total Number of Sampled Animals per Site	Number of Sampled Animals per Host Species	Number of RNA Positive Samples	Number of Antibody Positive Samples	Sex of RNA or Antibody Positive Animals
**Germany, Lower Saxony**	215				
Forestry Office Oerrel	119	CC (93)	-	3	1 Female, 2 Male
		CE (24)	-	1	Female
		DD (2)	-	-	
Forestry Office Unterlüß	63	CC (36)	-	1	Female
		CE (26)	-	-	
		DD (1)	-	-	
Forestry Office Wolfenbüttel	25	CC (24)	-	-	
		CE (1)	-	-	
Forestry Office Schulenburg	8	CC (7)	-	-	
		CE (1)	-	-	
**Czech Republic**	67				
Brno venkov	9	CE (4)	-	-	
		OM (5)	-	-	
Jičín	2	CC (2)	-	-	
Prostejov	7	CE (5)	-	-	
		DD (2)	-	-	
Chomutov	49	CC (12)	-	-	
		CE (19)	1	-	Male
		CN (16)	-	-	
		OM (2)	-	-	
